# Optimal Regimen of N-Acetylcysteine on Chromium-Induced Renal Cell Damage

**DOI:** 10.3390/metabo9090172

**Published:** 2019-08-28

**Authors:** I-Jeng Yeh, Tzu-Yi Wang, Jhong-Ching Lin, Tzeng-Jih Lin, Jung-San Chang, Meng-Chi Yen, Yao-Hua Liu, Pei-Lin Wu, Fen-Wei Chen, Yueh-Lun Shih, Chiung-Yu Peng

**Affiliations:** 1 Department of Emergency Medicine, Kaohsiung Medical University Hospital, Kaohsiung Medical University, Kaohsiung 807, Taiwan; 2 Family Medicine Department, Taoyuan Branch, Taipei Veterans General Hospital, Taipei 112, Taiwan; 3 Department of Renal Care, College of Medicine, Kaohsiung Medical University, Kaohsiung 807, Taiwan; 4 Division of Gastroenterology, Department of Internal Medicine, Kaohsiung Medical University Hospital, Kaohsiung Medical University, Kaohsiung 807, Taiwan; 5 Graduate Institute of Clinical Medicine, College of Medicine, Kaohsiung Medical University, Kaohsiung 807, Taiwan; 6 Department of Public Health, College of Health Science, Kaohsiung Medical University, Kaohsiung 807, Taiwan; 7 Research Center for Environmental Medicine, Kaohsiung Medical University, Kaohsiung 807, Taiwan

**Keywords:** apoptosis, hexavalent chromium, human proximal tubular epithelial cell, N-acetylcysteine (NAC), reactive oxygen species

## Abstract

Chromium (Cr) is a well-known heavy metal that can cause renal damage. The production of reactive oxygen species (ROS) due to chromium-induced toxicity induces cell dysfunction, apoptosis, and death. N-acetylcysteine (NAC) is an antioxidant used as an antidote for chromium-induced toxicity. However, the optimal regimen and protective mechanisms of NAC are not fully understood in human renal cells. Our results showed that exposure to 10 μM K_2_Cr_2_O_7_, a toxic Cr(VI) compound, induced apoptosis and production of intracellular ROS in the human proximal tubular epithelial cell line HK-2. Supplements of 600 or 1000 µg/mL NAC inhibited intracellular ROS in HK-2 cells exposed to Cr(VI) and significantly increased cell viability within 2 h of Cr(VI)-induced cytotoxicity. Moreover, Cr(VI) induced the expression of apoptosis markers, including cleaved-caspase-3, cleaved-poly (ADP-ribose) polymerase, cleaved-caspase 8, and cleaved-caspase 9, and altered the expression ratio of Bax/Bcl-xL. Expression of apoptosis markers within 2 h of Cr(VI)-induced cytotoxicity in cells treated with 600 µg/mL NAC was significantly suppressed. However, delayed treatment with NAC at 4 h and 8 h after exposure to Cr did not suppress the activation of apoptotic pathways. In summary, our study reports the optimum timing and dose of NAC for the protection of human renal proximal tubular cells from Cr(VI)-induced cell death. The NAC treatment strategy described could be applied in clinical practice to suppress renal cell apoptosis, which in turn could rescue renal function.

## 1. Introduction

Chromium (Cr) is an abundant element in the Earth’s crust. Trivalent Cr(III) and hexavalent Cr(VI) are the two most stable oxidative states of Cr in the natural environment. Compared to Cr(III), Cr(VI) is more cytotoxic because it can be absorbed by cells, which induces the production of reactive oxygen species (ROS) [1,2,3]. Cr(VI) has been detected in samples from various sources of water [4,5]. In addition, Cr(VI) exposure causes breaks in DNA strands in several types of mammalian cells [6,7,8]. Therefore, consuming Cr(VI)-contaminated water might be a risk factor for cancer development. Cr(VI) causes the disruption of metabolic regulation between carbohydrates and proteins in murine renal tissue [9]. Furthermore, common industrial applications of Cr(VI) increase the risk of acute occupational exposure to Cr(VI), which can lead to severe destruction of proximal renal tubular cells, resulting in significant deterioration of renal function in humans [10,11,12,13,14]. To manage the acute toxic effects of Cr(VI), hemodialysis [10], hemoperfusion [11], exchange transfusion [12,13], and peritoneal dialysis [14] were implemented in several clinical cases; however, these treatments did not provide any beneficial outcomes. Therefore, the development of an alternative and optimal strategy to manage the acute toxic effects of Cr(VI) remains an urgent issue.

When Cr(VI) is absorbed into cells it is rapidly reduced, subsequently resulting in the production of several reactive chromium intermediates, such as Cr(IV) and Cr(V), as well as ROS. All of these intermediates and molecules are considered to be responsible for altering the normal functions of cells and promoting apoptosis [15,16]. Based on its heavy metal properties and the oxidative stress it causes in cells, multiple chelating agents and antioxidant remedies have been proposed to rescue acute Cr-induced toxicity. Our recent study showed that an optimized dose of an antioxidant, l-ascorbic acid, protected human kidney cells from apoptosis [17]. N-acetylcysteine (NAC), a potent antioxidant remedy, has been safely and widely used to manage oxidative stress injury [18]. However, the mechanisms for cell rescue against the toxic effects of Cr have not yet been clearly elucidated. Because glutathione is a major endogenous antioxidant used for ROS elimination, and the absence of l-cysteine limits glutathione synthesis [19,20], the NAC-mediated protective effect on Cr toxicity might be due to the stimulation of glutathione synthesis [21,22,23]. In addition, NAC has been demonstrated as a chelator of some heavy metals, including lead, mercury, cadmium, and Cr [24]. Treatment of NAC via intraperitoneal injection could protect the kidneys and liver from Cr-induced oxidative stress in mice [25]. This evidence suggests that NAC could serve as a potential treatment for managing Cr-induced toxicity in clinical settings.

Currently, there is no direct evidence to support the hypothesis that NAC treatment can protect the kidneys against Cr damage. Furthermore, the regulatory mechanisms of NAC on Cr-induced apoptosis have not been determined in human renal cells. Thus, our study aims to investigate the optimal dosage of NAC, the optimal time-point of treatment after Cr-induced toxicity, the maximum delay-period for NAC to rescue Cr-induced apoptosis, and the possible molecular mechanisms of NAC on Cr-induced apoptosis in an immortalized human proximal tubular epithelial cell line.

## 2. Results

### 2.1. Cr(VI)-Induced Toxicity Results in HK-2 Apoptosis

**Figure 1:**
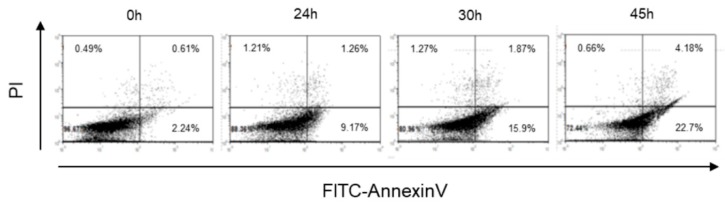
K_2_Cr_2_O_7_-induced apoptosis in HK-2 cells. Detection of apoptotic cells using Annexin V/propidium iodide (PI) at 24, 30, and 45 h.

To evaluate the toxic effects of Cr(VI), HK-2 cells were treated with potassium dichromate (K_2_Cr_2_O_7_) because it is a toxic compound of Cr(VI) and induces nephrotoxicity in humans and animals [26]. In our previous study, the cell viability of HK-2 significantly decreased after treatment with 10 μM of K_2_Cr_2_O_7_ [17]. The same concentration (10 μM) of K_2_Cr_2_O_7_ was used in this study. In Figure 1, the results of Annexin V/propidium iodide (PI) staining showed an increased number of apoptotic cells (Annexin V-positive/PI-negative cells) in K_2_Cr_2_O_7_-exposed groups when compared with those in the control group (0 h). The data indicated that 10 μM K_2_Cr_2_O_7_ induced apoptosis in the proximal tubular epithelial renal cell line HK-2.

### 2.2. NAC Treatment Protects HK-2 from Cr(VI)-Induced ROS

**Figure 2:**
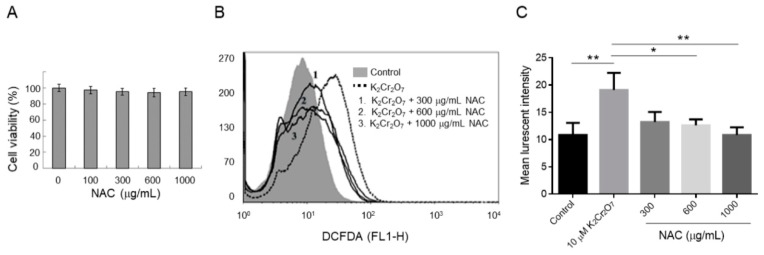
Effect of N-acetylcysteine (NAC) treatment on intracellular reactive oxygen species (ROS) of K_2_Cr_2_O_7_-induced cytotoxicity in HK-2 cells. (**A**) Cell viability at 24 h after exposure to different concentrations of NAC in HK-2 cells. (**B**) Detection of ROS levels by flow cytometry with different NAC in K_2_Cr_2_O_7_-treated HK-2 cells. The filled gray area indicates the control group and the dashed line indicates the 10 μM K_2_Cr_2_O_7_-treated group. The numbers 1, 2, and 3 indicate HK-2 cells exposed to 10 μM K_2_Cr_2_O_7_ and (1) 300 μg/mL NAC, (2) 600 μg/mL NAC, and (3) 1,000 μg/mL NAC. (**C**) Quantification of ROS levels. Data are presented as mean ± SD. * *P* < 0.05, ** *P* < 0.01, when compared with the 10 μM K_2_Cr_2_O_7_-treatment group.

To evaluate whether NAC induces a toxic effect, HK-2 cells were treated with different concentrations of NAC (from 0 to 1000 μg/mL). The viability of HK-2 cells was not significantly affected when the NAC concentration was lower than 1000 μg/mL (Figure 2A). Because ROS is a major cause of Cr(VI)-induced nephrotoxicity, the intracellular ROS levels in Cr(VI)-exposed HK-2 were evaluated by 2′,7′-Dichlorofluorescin diacetate (DCFDA) staining. The intracellular ROS levels were significantly higher in Cr(VI)-exposed cells than in control cells. The intracellular ROS levels of HK-2 cells showed a decreasing trend in the 300, 600, and 1000 μg/mL NAC-treated groups (Figure 2B). Moreover, the ROS level was significantly inhibited when the cells were co-treated with 600 and 1000 μg/mL NAC and 10 μM of K_2_Cr_2_O_7_ (Figure 2C).

### 2.3. NAC Treatment Protects HK-2 from Cr(VI)-Induced Cell Death

**Figure 3:**
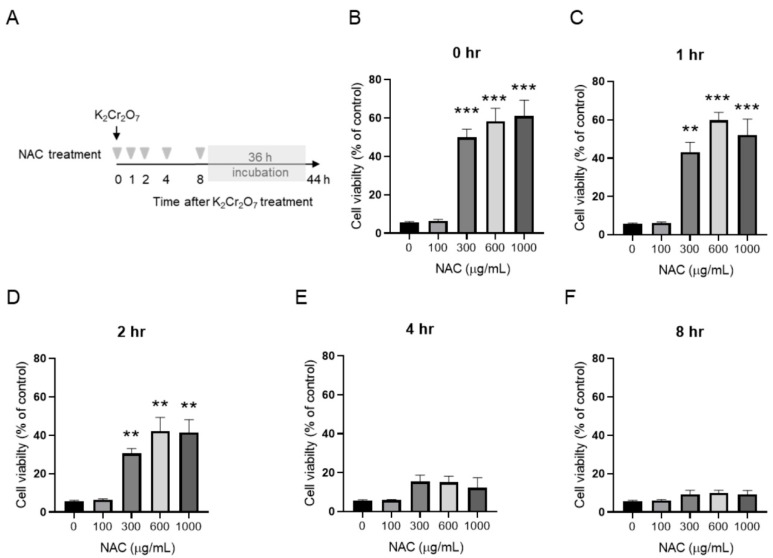
Protective effect of NAC on cell viability of HK-2 cells. (**A**) Scheme of time-delayed NAC treatment. The viability of HK-2 at (**B**) 0 h, (**C**) 1 h, (**D**) 2 h, (**E**) 4 h, and (**F**) 8 h post-treatment with 10 μM K_2_Cr_2_O_7_ is demonstrated. Data are presented as mean ± SD. * *P* < 0.05, ** *P* < 0.01, and *** *P* < 0.001, when compared with 0 g/mL NAC-treated group at each time-point.

**Figure 4:**
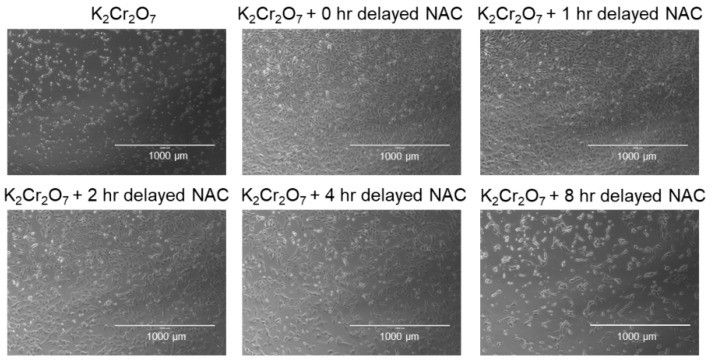
Morphology of HK-2 cells. (**A**) 10 μM K_2_Cr_2_O_7_-treated HK-2 cells and 10 μM K_2_Cr_2_O_7_-treated HK-2 cells with delayed NAC treatment at (**B**) 0 h, (**C**) 1 h, (**D**) 2 h, (**E**) 4 h, and (**F**) 8 h.

To further evaluate the protective effect of NAC on K_2_Cr_2_O_7_-induced toxicity, HK-2 cells was subjected to NAC treatment at different time-points post-K_2_Cr_2_O_7_ treatment (from 0 to 8 h) and then incubated for a further 36 h (Figure 3A). In Figure 3B–D, 300, 600, and 1000 μg/mL of NAC treatment significantly enhanced cell viability at 0, 1, and 2 h post-K_2_Cr_2_O_7_ treatment. In contrast, supplementation of NAC at 4 and 8 h post-K_2_Cr_2_O_7_ treatment (Figure 3E,F) had no benefits on the viability of HK-2 cells. The morphologies of NAC- and K_2_Cr_2_O_7_-treated HK-2 cells are shown in Figure 4. The regulation of the apoptotic pathway was examined further.

### 2.4. NAC Treatment Altered Cr(VI)-Induced Apoptotic Pathways

**Figure 5:**
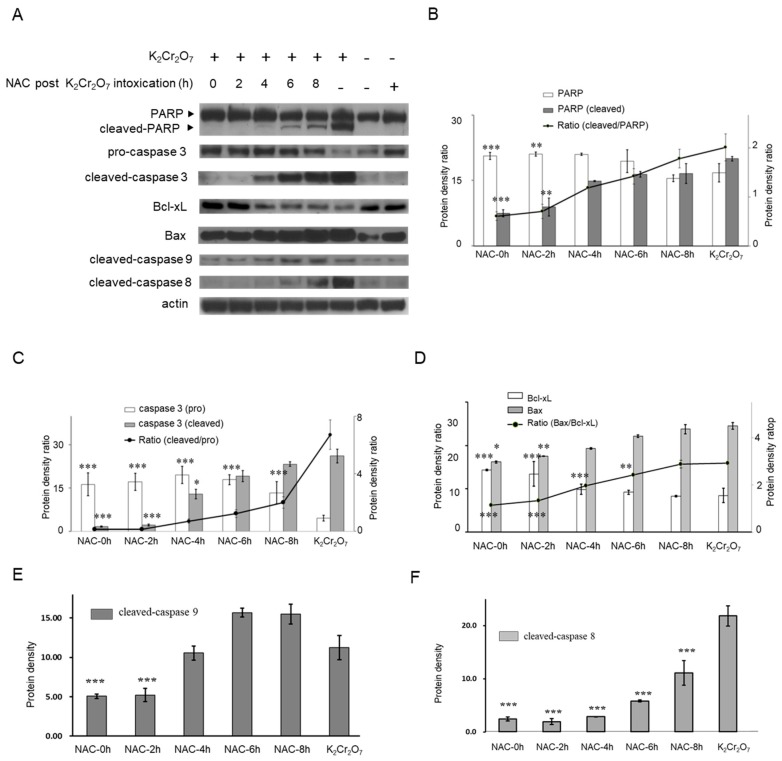
Evaluation of apoptosis signaling pathways. HK-2 cells were first exposed to 10 μM K_2_Cr_2_O_7_ treatment before being treated with NAC at 0, 2, 4, 6, and 8 h. Protein lysates were collected after further incubation at 36 h. (**A**) Evaluation of the protective effect of NAC on K_2_Cr_2_O_7_-induced apoptosis. The levels of expression of poly (ADP-ribose) polymerase (PARP), cleaved-PARP, pro-caspase 3, cleaved-caspase 3, Bax, Bcl-xL, cleaved-caspase 9, and cleaved-caspase 8 were determined at different time-points of NAC treatment. Quantitative results of (**B**) PARP (*n* = 3), (**C**) caspase 3 (*n* = 3), (**D**) Bax/Bcl-xL ratio (*n* = 3), (**E**) cleaved-caspase 9 (*n* = 3), and (**F**) cleaved-caspase 8 (*n* = 3) are shown. Data in the bar plots are presented as mean ± SD. * *P* < 0.05, ** *P* < 0.01, *** *P* < 0.001 when compared with 10 μM K_2_Cr_2_O_7_-treatment group.

**Figure 6:**
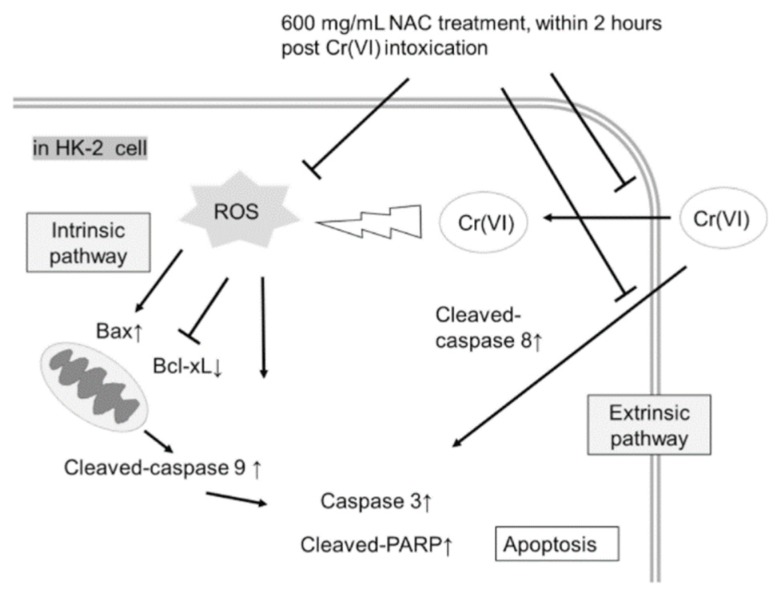
Proposed scheme of NAC treatment. Cr(VI) exposure induced ROS and then activated apoptotic pathways in human epithelial renal proximal tubule cells. In addition, extrinsic pathways were induced by an unknown mechanism. NAC treatment within 2 h post Cr(VI)-induced toxicity effectively rescued cells from apoptosis.

The results of the Western blot assay revealed that expression of apoptotic markers, including cleaved-poly (ADP ribose) polymerase (PARP) and cleaved-caspase 3 was induced after K_2_Cr_2_O_7_ exposure (Figure 5), in addition to the ratio of Bax/Bcl-xL and cleaved-caspase 9 expression. In contrast, NAC treatment of HK-2 cells did not significantly induce expression levels of cleaved-PARP and cleaved-caspase 3 compared to those in HK-2 control cells. Since the protective effects of NAC were demonstrated at certain time-points (as depicted in Figure 3 and Figure 4), the statuses of PARP, caspase 3, Bax, Bcl-xL, caspase 9, and caspase 8 were also evaluated at the same time-points. Our results showed that the cleaved-PARP and cleaved-caspase 3 protein levels were almost inhibited at 0 and 2 h post-K_2_Cr_2_O_7_ treatment. In addition, a relatively low ratio of Bax/Bcl-xL and inhibition of caspase 9 activaty were observed at 0 and 2 h post-K_2_Cr_2_O_7_-induced toxicity. In contrast, the NAC treatment significantly inhibited the activation of caspase 8 up to 8 h post-K_2_Cr_2_O_7_ treatment (Figure 5E). The summarized graph of the present study is presented in Figure 6.

## 3. Discussion

Chromium induces the production of free radicals by a Fenton-type reaction, a Haber–Weiss reaction, or by reacting directly with cellular molecules, triggering multiple apoptosis-signaling pathways in several cell types [27,28,29]. A chromium concentration in of 10 mg/L or higher in the blood is considered to be a lethal dose for humans [30]. Other reports described that a chromium blood concentration of 3.4 mg/L induced acute renal dysfunction without leading to a lethal result [31]. In this study, HK-2 cells exposed to 10 µM (2.95 mg/L) of Cr(VI) were at an increased likelihood of cell death, with higher intracellular ROS levels detected (Figure 1 and Figure 2). Our results further demonstrated that co-administration of NAC and Cr(VI) resulted in the suppression of intracellular ROS production, suggesting a satisfactory efficacy of NAC in the prevention of chromium-induced renal damage by means of its chelating effect [24,32,33].

NAC has been widely used in renal protection against oxidative stress injury, such as ischemia-reperfusion injury [34,35], nephrotoxin-induced injury [36,37,38], and chronic kidney disease [39], thus suggesting that it is a safe and effective treatment for oxidative stress injuries. The optimal dose and tolerable lag period of NAC treatment was evaluated in HK-2 cells. Our results revealed that a supplement with 600 µg/mL NAC did not induce cytotoxicity and significantly inhibited intracellular ROS in human epithelial renal proximal tubule cells. In addition, NAC treatment significantly enhanced cell viability when cells were treated with 600 µg/mL NAC within 2 h of Cr(VI)-induced toxicity (Figure 3 and Figure 4). In a Cr(VI)-treated mice model, treatment with NAC (200 mg/kg, intraperitoneal injection) an hour before Cr(VI) (20 mg/kg) treatment and an hour after Cr(VI) toxicity provided beneficial effects in the liver and kidney tissue [25]. Clinical data previously showed that administration of 150 mg/kg of NAC for the treatment of acetaminophen overdose led to a mean maximum concentration of NAC of 554 µg/mL in plasma [40]. Based on the above evidence, 150–200 mg/kg NAC may be an optimal range of dosage in the treatment of Cr(VI)-induced renal toxicity. Moreover, our results suggested that the tolerable time gap for NAC treatment is less than 2 h. This finding may explain why a supplement of 50 mg/kg NAC after 6 h of Cr exposure could not prevent the development of life-threatening phenomena [41].

Current evidence suggests that Cr(VI)-induced toxicity brings about cell apoptosis, mainly via intrinsic mitochondrial pathways but not extrinsic pathways, in several types of cells, including human lung tumor cells, lymphoma cells, anterior pituitary cells, hepatocyte cells, and colon carcinoma cells [15,42,43]. The intrinsic pathway is regulated by pro-apoptotic and anti-apoptotic proteins, such as Bax and Bcl-xL, respectively [44]. In contrast, caspase 8 plays a critical role in the extrinsic apoptotic signaling pathway [44]. Figure 5 displays our results demonstrating that NAC treatment within 2 h of Cr(VI)-induced toxicity significantly decreased the ratio of Bax/Bcl-xL and the activation of caspase 9, which implied that the intrinsic apoptosis pathways were inhibited. In addition, delayed treatment with NAC, up to 8 h, could only block the extrinsic pathway (cleaved-caspase 8). These results implied that Cr(VI) first triggers the intrinsic pathways, followed thereafter by the extrinsic pathways. Because our results demonstrated that HK-2 cells had to be treated with NAC within 2 h of Cr(VI)-induced toxicity to reduce damage, we inferred that the therapeutic effect of NAC was mainly dependent on inhibition of intrinsic pathways in these cells. We supposed that the delay and inhibition of only the extrinsic pathways may be insufficient to block apoptosis. Further experimental evidence is needed to support this hypothesis. For example, z-IETD-FMK, a specific caspase-8 inhibitor, can be used to investigate the importance of extrinsic pathways in Cr(VI)-induced cell death or NAC protection. We will perform these experiments in the future.

It is interesting to note that the Bax level in NAC increased when compared to untreated cells (Figure 5A). A high concentration (2 to 5 mM) of NAC treatment can induce apoptosis in some types of cells, such as vascular smooth muscle cells and myoblastic cells, alongside an increase in Bax expression levels [45,46]. In this study, the cell viability of HK-2 was not significantly affected after NAC treatments at concentrations of 100 μg/mL to 1000 μg/mL (0.613 to 6.13 mM). Therefore, the current data suggest that Bax was also induced by NAC, but cell viability was not affected. The role of Bax in NAC-treated renal cells needs to be further investigated in the future.

There are some limitations to this study. The optimal doses of NAC and periods of delayed NAC treatment were evaluated in a single cell line in vitro, but not evaluated in vivo. Therefore, further detailed studies on this treatment are still required in the future.

## 4. Conclusions

Our observations are the first to describe the optimal timing and dose of NAC in the protection of human renal proximal tubular cells from Cr(VI)-induced cell death. Our results imply that the strategy of NAC treatment could be applied in clinical practice, because inhibition of apoptosis might rescue renal function. These results could help in the design of an NAC treatment strategy, which could provide novel evidence to assist emergency physicians in the treatment of curable, but not lethal, chromium toxicity.

## 5. Materials and Methods

### 5.1. HK-2 Cell Culture

HK-2 cells (ATCC CRL-2190) derived from an adult human normal kidney were characterized as a proximal tubular epithelial renal cell line [47]. HK-2 was purchased from American Type Culture Collection (ATCC) and maintained in keratinocyte-serum-free medium (K-SFM) supplemented with bovine pituitary extract (BPE), human recombinant epidermal growth factor (EGF), and 1% penicillin–streptomycin (Life Technologies, CA, USA). Cells were incubated at 37 °C in a humidified atmosphere containing 95% air and 5% CO_2_.

### 5.2. MTT Assay for Cell Viability

To determine the toxicity of NAC, 1 × 10^4^ HK-2 cells were treated with various concentrations (100, 300, 600, and 1000 µg/mL) of NAC (A8199, Sigma-Aldrich, Saint Louis, USA) for 24 h. To determine the protective effect of NAC, 1 × 10^4^ HK-2 cells were treated with 10 µM K_2_Cr_2_O_7_ and various concentrations of NAC (100, 300, 600, and 1000 µg/mL) at different time-points after chromium exposure (0, 1, 2, 4, and 8 h). After incubating further for 24 h, cell viabilities were directly examined by an inverted microscope, Eclipse Ti-U (Nikon, Tokyo, Japan), under 400× *g* magnification, and indirectly assayed using a 3-(4,5-dimethylthiazol-2-yl)-2,5-diphenyl-tetrazolium bromide (MTT) kit (Sigma-Aldrich, Schnelldorf, Germany), according to the manufacturer’s instruction. The absorbance at A570 nm was determined by an ELISA reader (Multiskan EX, Labsystems, MA, USA).

### 5.3. Annexin V/Propidium Iodide (PI) Staining

The apoptosis phenotype was analyzed by fluorescein (FITC)-conjugated Annexin V and a propidium iodide detection kit (BD Technologies, New Jersey, USA). HK-2 1 × 10^6^ cells were seeded into 10 cm culture dishes with serum free Dulbecco’s Modified Eagle’s Medium (DMEM) (Sigma-Aldrich, Saint Louis, USA). K_2_Cr_2_O_7_ (10 μM) was added to the cells and subsequently cultured for 24, 30, and 45 h. After removing the supernatant, which included the dead/suspended cells, attached cells were harvested, washed with cold PBS, suspended in 100 μL Annexin V-FITC binding buffer and 5 μL Annexin V-FITC or 5 μL propidium iodide (PI), and incubated at room temperature in the dark for 15 min. The samples were analyzed via a Partec Cyflow machine (Sysmex Partec GmbH, Gorlitz, Germany). The results were determined according to a four-quadrant diagram and analyzed using FlowJo software (Leonard Herzenberg, NY, USA).

### 5.4. Oxidative Stress Assays

The production of reactive oxygen species (ROS) as a result of chromium toxicity was detected by flow cytometry. HK-2 cells (1 × 10^6^) were incubated in 10 cm culture dishes with 5 µM 2′7′-dichlorofluorescein diacetate (H2DCFDA) (Sigma-Aldrich, Schnelldorf, Germany) at 37 °C for 30 min. After centrifugation and washing with PBS, HK-2 cells were exposed to 10 µM K_2_Cr_2_O_7_ and supplemented with various concentrations of NAC (300 µg/mL, 600 µg/mL, and 1000 µg/mL) in triplicate. Following 30 min of incubation, fluorescence intensity which correlates with hydroxyl radical concentration was detected by Partec CyFlow (Partec, Münster, Germany). Data were analyzed by FCS Express 4 Flow Cytometry (De Novo, Los Angeles, CA, USA). All procedures were performed on ice with protection from light.

### 5.5. Apoptotic Assay by Western Blot Analysis

HK-2 cells (10^6^) were seeded into 10 cm culture dishes and cultured with K-SFM and supplemented until 80% confluence was reached. Cells were washed with PBS twice and K-SFM was replaced with serum-free DMEM. After treatment with 10 µM K_2_Cr_2_O_7_, 600 µg/mL NAC was supplemented at 0, 2, 4, 6, and 8 h, and the cells were further incubated for 36 h. After washing with cold PBS, cells were supplemented with 200 μL radioimmunoprecipitation (RIPA) lysis buffer (Amresco, Ohio, USA) containing 1% proteinase inhibitor and kept on ice for 20 min. Total cellular proteins were extracted by gently vortexing and centrifuging at 16,000 g for 20 min at 4 °C. To extract the cytosolic protein fraction, the Nuclear Protein Isolation-Translocation Assay Kit (FIVEphoton Biochemical, San Diego, USA) was used. Proteins were quantified using Bio-Rad protein assay kit (Bio-Rad, CA, USA). Later, total proteins (40 μg) were mixed with sample buffer (5% mercaptoethanol, 0.02% bromophenol blue, 30% glycerol, 10% sodium dodecyl sulfate (SDS), 250 mM pH 6.8) and then boiled for 5–10 min at 95 °C. The mixture was separated on a 10% SDS–polyacrylamide gel and electrophoresis was performed at 100 V for 1 h. The separated proteins were transferred to a Hybond-P polyvinylidene difluoride (PVDF) membrane (Amersham Biosciences, UK) and blocked with 3% bovine serum albumin (BSA) in Tris-buffered saline containing Tween 20 (TBST) for 1 h. Apoptosis-related proteins were detected by a 1:1000 dilution of commercial monoclonal antibodies, including primary antibodies for the detection of anti-poly (ADP-ribose) polymerase (PARP) (1:1000, Cell Signaling Technology, MA, USA), pro-caspase 3 (1:1000, Millpore, Temecula, CA, USA), cleaved-caspase 3 (1:,000, Sigma, Temecula, CA, USA), Bcl-xL (1:1000, Biolegend, San Diego, CA, USA), Bax (1:1000, Cell Signaling Technology, MA, USA), cleaved-caspase 9 (1:1000, Cell Signaling Technology, MA, USA), and cleaved-caspase 8 (1:1000, Cell Signaling Technology, MA, USA) overnight at 4 °C. This was followed by a 1:5000 dilution of goat anti-mouse IgG- horseradish peroxidase (HRP) conjugated antibody (Biolegend, San Diego, USA) or a 1:10,000 dilution goat anti-rabbit IgG-HRP conjugated antibody (Biolegend, san Diego, USA) at room temperature for 1 h. The internal control, β-actin (Chemicon, CA, USA), was assayed using a 1:1000 dilution of primary antibody and detected by the same secondary antibody described above. Target proteins were visualized with Clarity™ Western enhanced luminol-based chemiluminescent substrate (ECL) Substrate (Bio-Rad, CA, USA) and HyBlot CL film (Denville Scientific Inc, NJ, USA). The density of each band was quantified with ImageJ software (NIH, Maryland, USA).

### 5.6. Statistical Analysis

The results of at least three independent experiments were expressed as mean ± SD. Data were analyzed using ANOVA by SPSS20 software (SPSS, Chicago, USA). Scheffe’s test was used for post-hoc analysis to compare all pairs of the groups in the ANOVA test. The level of significance was set at *P* < 0.05.
